# The Pathobiology of TDP-43 C-Terminal Fragments in ALS and FTLD

**DOI:** 10.3389/fnins.2019.00335

**Published:** 2019-04-11

**Authors:** Britt A. Berning, Adam K. Walker

**Affiliations:** ^1^Neurodegeneration Pathobiology Laboratory, Queensland Brain Institute, University of Queensland, Brisbane, QLD, Australia; ^2^Centre for Motor Neuron Disease Research, Department of Biomedical Sciences, Faculty of Medicine and Health Sciences, Macquarie University, North Ryde, NSW, Australia

**Keywords:** amyotrophic lateral sclerosis (ALS), motor neuron disease (MND), TDP-43, neurodegeneration, frontotemporal lobar degeneration (FTLD-TDP)

## Abstract

During neurodegenerative disease, the multifunctional RNA-binding protein TDP-43 undergoes a vast array of post-translational modifications, including phosphorylation, acetylation, and cleavage. Many of these alterations may directly contribute to the pathogenesis of TDP-43 proteinopathies, which include most forms of amyotrophic lateral sclerosis (ALS) and approximately half of all frontotemporal dementia, pathologically identified as frontotemporal lobar degeneration (FTLD) with TDP-43 pathology. However, the relative contributions of the various TDP-43 post-translational modifications to disease remain unclear, and indeed some may be secondary epiphenomena rather than disease-causative. It is therefore critical to determine the involvement of each modification in disease processes to allow the design of targeted treatments. In particular, TDP-43 C-terminal fragments (CTFs) accumulate in the brains of people with ALS and FTLD and are therefore described as a neuropathological signature of these diseases. Remarkably, these TDP-43 CTFs are rarely observed in the spinal cord, even in ALS which involves dramatic degeneration of spinal motor neurons. Therefore, TDP-43 CTFs are not produced non-specifically in the course of all forms of TDP-43-related neurodegeneration, but rather variably arise due to additional factors influenced by regional heterogeneity in the central nervous system. In this review, we summarize how TDP-43 CTFs are generated and degraded by cells, and critique evidence from studies of TDP-43 CTF pathology in human disease tissues, as well as cell and animal models, to analyze the pathophysiological relevance of TDP-43 CTFs to ALS and FTLD. Numerous studies now indicate that, although TDP-43 CTFs are prevalent in ALS and FTLD brains, disease-related pathology is only variably reproduced in TDP-43 CTF cell culture models. Furthermore, TDP-43 CTF expression in both transgenic and viral-mediated *in vivo* models largely fails to induce motor or behavioral dysfunction reminiscent of human disease. We therefore conclude that although TDP-43 CTFs are a hallmark of TDP-43-related neurodegeneration in the brain, they are not a primary cause of ALS or FTLD.

## Introduction

Carboxyl-terminal fragments (CTFs) of *trans* active response DNA binding protein of 43 kDa (TDP-43) are frequently detected in the brains of people with amyotrophic lateral sclerosis (ALS) and frontotemporal lobar degeneration (FTLD) (Neumann et al., 2006). TDP-43 CTFs are therefore largely considered a hallmark of these diseases. However, TDP-43 CTFs are rarely detected in the characteristic pathological TDP-43 inclusions of motor neurons in the ALS spinal cord (Igaz et al., 2008), and exogenous expression of these fragments in cells and small animal models only variably recapitulates aspects of ALS and FTLD. In this review, we summarize how TDP-43 CTFs are formed and degraded and highlight how differences in the ability of distinct cell populations to generate or degrade TDP-43 CTFs may account for the regional variation in CTF levels in the brain and spinal cord in disease. We also critique evidence from human post-mortem tissue as well as cell culture and animal models of ALS and FTLD to conclude that, although TDP-43 CTFs are closely associated with disease pathology, they do not appear to play a causal role in disease onset or progression. The intention of this critique is to shift research attention toward molecular events that play a clear, causal role in the etiology of ALS and FTLD in order to improve the design of treatments targeted at disease-relevant dysfunctions related to TDP-43.

### TDP-43 Biology

TDP-43 is a 414-amino acid protein encoded by the *TARDBP* gene. It is ubiquitously expressed and predominantly locates to the nucleus owing to a nuclear localization signal (NLS) (Winton et al., 2008). TDP-43 also contains a nuclear export sequence that facilitates cytoplasmic shuttling (Ayala et al., 2008), although more recent work suggests that TDP-43 may also passively diffuse between the nucleus and cytoplasm (Ederle et al., 2018; Pinarbasi et al., 2018). The domain architecture of TDP-43 is similar to that of other RNA binding proteins, including an N-terminal domain with two highly conserved RNA recognition motifs (RRM1 and RRM2), each approximately 60 residues long, that preferentially bind RNA with UG repeat sequences (Buratti and Baralle, 2001). TDP-43 targets a wide range of RNA transcripts, including pre-mRNA, microRNA, and long intronic sequences (Polymenidou et al., 2011; Tollervey et al., 2011; Xiao et al., 2011; Colombrita et al., 2012; Narayanan et al., 2013). This enables TDP-43 to critically regulate a multitude of RNA processing pathways, such as alternative splicing, transcriptional repression, RNA stability, and microRNA biogenesis (Mercado et al., 2005; Kawahara and Mieda-Sato, 2012). TDP-43 also tightly regulates its own transcription via a negative feedback loop that maintains consistent protein levels; by binding to the 3′ untranslated region (UTR) of its own messenger RNA (mRNA), TDP-43 promotes degradation of the *TARDBP* transcript (Ayala Y. M. et al., 2011). This exquisite transcriptional control is demonstrated by the finding that TDP-43 protein levels do not differ between heterozygous *Tardbp* knockout mice and wild-type controls (Sephton et al., 2010). This self-regulatory function is essential to cell survival as both depletion and overexpression of TDP-43 are toxic *in vivo* and in cultured cells (Johnson et al., 2008; Kraemer et al., 2010; Voigt et al., 2010; Wu et al., 2010).

In addition to the direct RNA-binding functions of TDP-43, the glycine-rich C-terminal domain of TDP-43 mediates protein-protein interactions (Buratti et al., 2005; Buratti and Baralle, 2008; Freibaum et al., 2010; Uversky, 2015; Santamaria et al., 2017), which likely play additional roles in TDP-43 biochemistry. *In silico* analysis predicts that the C-terminal tail is inherently disordered and of low complexity (Bryson et al., 2005). This region of the TDP-43 protein mediates liquid–liquid phase separation (Conicella et al., 2016; Li et al., 2018a,b), a necessary step in the formation of membraneless organelles such as stress granules (Liu-Yesucevitz et al., 2010; Molliex et al., 2015; Mackenzie et al., 2017; Santamaria et al., 2017; Uversky, 2017; Fang et al., 2018). TDP-43 also undergoes a myriad of post-translational modifications, including ubiquitination, phosphorylation, and acetylation, that alter its structure and biochemical function (for reviews, see Sambataro and Pennuto, 2017; Buratti, 2018).

### ALS and FTLD Disease and TDP-43 Pathology

Amyotrophic lateral sclerosis is a fatal neurodegenerative disorder that is typically adult-onset, and is characterized by progressive loss of upper motor neurons in the motor cortex and corticospinal tract, and lower motor neurons in the spinal cord (Robberecht and Philips, 2013). This leads to denervation and rapid atrophy of specific muscle groups, which usually eventuates in death by respiratory failure (Neudert et al., 2001). Over 97% of ALS cases, both sporadic and familial, feature TDP-43-positive inclusions in the cytoplasm of affected neurons (Ling et al., 2013). In addition, TDP-43 inclusions in frontal and anterior temporal lobe regions are the hallmark of some forms of frontotemporal dementia (FTD). Rather than affecting the motor system, in FTD these inclusions are associated with the progressive development of deficits in behavior, affect and language processing with relative sparing of memory and visuo-spatial skills, at least early in disease (Kirshner, 2014; Bang et al., 2015). At post-mortem, this form of FTD is classified as FTLD with TDP-43 pathology (FTLD-TDP), and accounts for approximately half of FTD cases, distinguished from cases in which pathological tau, fused in sarcoma (FUS) or other proteins predominate (Ling et al., 2013).

In ALS and FTLD patients, surviving neurons with TDP-43 pathology typically show accumulation of TDP-43 in cytoplasmic inclusions, and clearance of nuclear TDP-43, suggesting that TDP-43 pathogenicity may proceed via both loss of normal function as well as toxic gains of function. In disease, TDP-43 inclusions have varying morphologies, ranging from neuronal cytoplasmic inclusions with compact rounded or skein-like appearance, to short or long dystrophic neurites, to rare intranuclear inclusions or glial (primarily oligodendrocytic) inclusions (Neumann et al., 2006, 2009). The presence of various forms of TDP-43 pathology in both ALS and FTLD suggests that these diseases may occur as manifestations of a spectrum of TDP-43-related disease (Geser et al., 2010; Ling et al., 2013). Furthermore, as disease advances, many people living with ALS develop impairments in letter fluency, executive function and behavior that, to a degree, resemble behavioral-variant FTD (Crockford et al., 2018). However, recent neuropathological findings suggest that FTLD cases present with distinct forms of TDP-43 pathology in comparison with ALS and FTLD-ALS cases, indicating divergent mechanisms of disease pathogenesis that nonetheless involve the same protein, TDP-43 (Tan et al., 2017b). Further research is therefore required to understand how and why unique TDP-43 pathology develops in distinct diseases, as well as in different subtypes of one disease.

Based on the distribution and morphology of TDP-43 species, FTLD-TDP pathology can be stratified into at least four subtypes, which partially correlate with the underlying genetics and clinical presentation (Mackenzie et al., 2011; Van Mossevelde et al., 2018). In updated consensus nomenclature, the two most common subtypes are FTLD-TDP type A, which is defined by the presence of many neuronal cytoplasmic TDP-43 inclusions and short dystrophic neurites primarily in cortical layer 2, and FTLD-TDP type B, which is defined by many cytoplasmic TDP-43 inclusions that are distributed throughout all cortical layers, with few dystrophic neurites (Mackenzie et al., 2011; Tan et al., 2013). The rarer FTLD-TDP type C pathology presents with few cytoplasmic TDP-43 inclusions but abundant long dystrophic neurites primarily in cortical layer 2, and the very rare FTLD-TDP type D pathology, linked with mutations in the valosin-containing protein gene (*VCP*), has few cytoplasmic TDP-43 inclusions but many lentiform neuronal intranuclear inclusions throughout the cortical layers (Mackenzie et al., 2011; Tan et al., 2013). Additional subtypes of TDP-43 pathology have also been suggested, such as rapidly progressive FTD cases with widespread TDP-43 pathology and distinct patterns of TDP-43 CTFs (termed ‘type E,’ Lee E. B. et al., 2017), although the wider prevalence of this form of disease is unclear. Recent work has shown that FTLD-TDP subtypes may also be distinguished according to the classes of proteins that become insoluble in addition to TDP-43 (Laferriere et al., 2019).

Although underlying TDP-43 pathology does not always correlate with genetics or disease phenotype, mutations to the progranulin (*GRN*) gene are generally associated with type A TDP-43 pathology, whereas hexanucleotide repeat expansions in *C9orf72*, which is the most common genetic cause of ALS and FTLD-TDP, most frequently result in type B pathology (Mackenzie et al., 2011; Boeve et al., 2012). Patients who have developed both FTLD and ALS disease features also tend to exhibit type B TDP-43 pathology in the brain at autopsy (Mackenzie et al., 2011). Overall, the involvement of TDP-43 pathology in different forms of disease indicates the key role that TDP-43 plays in pathophysiology. The large variety of genetic factors, inclusion morphologies and clinical symptoms related to TDP-43 suggests that numerous contributing biochemical mechanisms likely converge on common pathogenic pathways in disease.

### TDP-43 and Neurodegenerative Disorders

Pathological TDP-43 is also evident in approximately 30–70% of Alzheimer’s disease patients, particularly those with fast progressing disease and advanced β-amyloid plaque and neurofibrillary tangle pathology (Josephs et al., 2016), and the presence of TDP-43 pathology is associated with mutation to the *APOE ε4* gene independent of β-amyloid pathology (Wennberg et al., 2018). TDP-43 pathology is observed in Parkinson’s Disease (Nakashima-Yasuda et al., 2007) and Huntington’s Disease (Schwab et al., 2008), and accumulates in astrocytes and white matter in the brains of patients with the rare neurodegenerative disorder Alexander disease (Walker et al., 2014). Phosphorylated TDP-43 was also associated with cognitive decline related to aging (Wilson et al., 2019). The presence of abnormal TDP-43 in numerous neurodegenerative disorders suggests that it may be a point of convergence in cellular dysfunction and death, despite diverse upstream disease mechanisms.

## TDP-43 CTFs Are Characteristic of Disease Pathology in Brain but Are Rarely Detected in Spinal Cord

**FIGURE 1:**
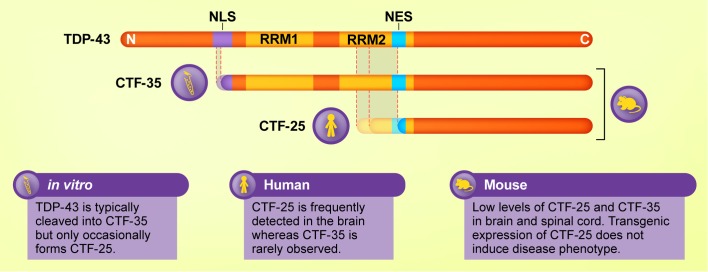
Schematic diagram of major TDP-43 C-terminal fragments (CTFs) and their cleavage sites and functional domains, and the experimental models in which they are most commonly detected. Although CTF-35 is frequently formed in cellular and *in vitro* assays of TDP-43 proteinopathies, this fragment is rarely observed in ALS or FTLD brain tissue, where CTF-25 predominates. Mouse models of TDP-43 exhibit low levels of both fragments. Thus, cleavage of TDP-43 varies according to model and species.

Examination of post-mortem ALS and FTLD-TDP brain tissue reveals the presence of low molecular weight TDP-43 species in addition to the full-length protein in detergent-insoluble protein fractions, which contain aggregated proteins, regardless of whether the person had ALS or FTLD (Neumann et al., 2006; Igaz et al., 2008). Immunoblotting using antibodies targeting the TDP-43 C-terminal domain consistently detects one or multiple fragments from the C-terminal portion of TDP-43 of approximately 25 kDa in the frontal and temporal lobes of people with ALS and FTLD. Henceforth we refer to these TDP-43 CTFs as CTF-25 (see Figure 1). CTFs of approximately 15 and 35 kDa (CTF-35) have also been detected in ALS and FTLD nervous tissue (Tsuji et al., 2012b; Xiao et al., 2015), although much less frequently and with inconsistency between studies (Lee et al., 2011). Evidence for the presence of TDP-43 CTFs in ALS or FTLD post-mortem tissue is summarized in Table 1. For consistency, we have re-categorized the results from early FTLD studies that used alternative nomenclature relating to the previous conflicting FTLD-U nomenclature systems (Mackenzie et al., 2006; Sampathu et al., 2006) according to the more recent consensus FTLD-TDP subtyping (Mackenzie et al., 2011).

**Table 1:** Summary of studies examining the presence of TDP-43 CTF-25 in postmortem brain and spinal cord tissue from people with ALS and FTLD-TDP.

Disease		Tissue Type	Presence of CTFs	Major Findings
ALS		Brain	+	• Increased CTFs compared to healthy controls in frontal and temporal cortices (Hasegawa et al., 2008; Tsuji et al., 2012a; Tsuji et al., 2012b; Kwong et al., 2014; Smethurst et al., 2016), hippocampus, substantia nigra, striatum, medulla and pons (Tsuji et al., 2012a), and in studies where brain region was not specified (Inukai et al., 2008; Zhang et al., 2009; Kametani et al., 2016)
			-	• No CTFs in temporal cortex or cerebellum (Neumann et al., 2006; Tsuji et al., 2012a)
				• Low levels of CTFs in the frontal cortex of some patients (Neumann et al., 2006)
		Spinal Cord	?	• CTFs faintly detected in the lumbar spinal cord, less abundant than full-length TDP-43 (Tsuji et al., 2012a; Smethurst et al., 2016)
				• Low levels of CTFs in both ALS and healthy control tissue (Giordana et al., 2010)
				• CTFs observed in one patient with a *TARDBP* mutation (Q343R) but not in sporadic ALS patients (Yokoseki et al., 2008)
				• 35 kDa fragment was detected in the ALS spinal cord (Xiao et al., 2015)
			-	• No CTFs (immunoblotting) in lumbar spinal cord (Neumann et al., 2006, 2009; Igaz et al., 2008; Uchida et al., 2012)
				• No CTFs (immunohistochemistry) in spinal cord ventral horn (Igaz et al., 2008; Kwong et al., 2014)
FTLD-TDP	Type A	Brain	+	• CTFs detected in cortex (Neumann et al., 2006; Igaz et al., 2008; Arai et al., 2010; Tsuji et al., 2012a,b; Lee E. B. et al., 2017; Porta et al., 2018) and hippocampus (Neumann et al., 2006)
	Type B	Brain	+	• CTFs detected in cortex (Igaz et al., 2008; Arai et al., 2010; Tsuji et al., 2012a,b; Lee E. B. et al., 2017; Porta et al., 2018)
	Type C	Brain	+	• CTFs detected in cortex (Neumann et al., 2006, 2007; Igaz et al., 2008; Arai et al., 2010; Hasegawa et al., 2011; Tsuji et al., 2012a,b; Lee E. B. et al., 2017; Watanabe et al., 2018; Porta et al., 2018), hippocampus (Neumann et al., 2006; Tsuji et al., 2012a) and striatum (Tsuji et al., 2012a)
			-	• No CTFs in cerebellum, substantia nigra, pons, medulla or thalamus (Tsuji et al., 2012a)
	Type D	Brain	?	• Low levels of CTFs variably detected in frontal and temporal cortices (Neumann et al., 2007)
	Type E	Brain	+	• Multiple CTFs between 23 and 26 kDa detected in frontal cortex (Lee E. B. et al., 2017)


*“+” indicates overt presence, “-” indicates no presence and “?’ indicates very low level detection or unclear findings.*

Immunoblotting using antibodies that specifically recognize phosphorylated epitopes (notably serines S403, S404, S409, S410) in the C-terminal domain of TDP-43 consistently detects CTF-25 in the brains of both FTLD-TDP and ALS patients (Hasegawa et al., 2008; Inukai et al., 2008; Neumann et al., 2009; Arai et al., 2010; Tsuji et al., 2012a,b, Kametani et al., 2016; Watanabe et al., 2018). Although these antibodies typically demonstrate a greater abundance of CTF-25 than full-length TDP-43 in the detergent-insoluble protein fraction (Tsuji et al., 2012a,b), the relative abundance of TDP-43 CTFs in relation to full-length TDP-43 varies according to the specific TDP-43 antibody used, with other studies reporting relatively low levels of CTF-25 (Arai et al., 2006; Neumann et al., 2006). Upon phosphatase treatment, the ∼25 kDa band detected by immunoblotting appears as multiple bands ranging from 18 to 27 kDa in size (Neumann et al., 2006). N-terminal sequencing of TDP-43 CTFs purified from FTLD-TDP brains has revealed a CTF-25 with a cleavage site at arginine residue 208 (Igaz et al., 2009) or asparagine residues 219 or 247 (Nonaka et al., 2009). A 32–35 kDa CTF has also been predicted to arise from cleavage at residue 89 (Zhang et al., 2007) and high throughput analysis of ALS brains indicates numerous other cleavage sites (Nonaka et al., 2009; Kametani et al., 2016).

The N-terminal portion resulting from TDP-43 cleavage has proven difficult to detect experimentally in human autopsy tissues (Igaz et al., 2008; Kwong et al., 2014), although other studies have suggested the presence of ∼32 kDa and ∼35 kDa truncated N-terminal TDP-43 proteins resulting from cleavage by asparaginyl endopeptidase (Herskowitz et al., 2012) and calpains (Yamashita et al., 2012, 2016). However, it has been suggested that any resultant N-terminal fragment of TDP-43 is likely rapidly degraded following cleavage of the full-length protein (Pesiridis et al., 2011; Buratti, 2018).

Although fragments of the C-terminal portion of TDP-43 are often regarded as a molecular signature of TDP-43 proteinopathies, the presence of TDP-43 CTFs does not necessarily correlate with neurodegeneration. Despite the post-mortem evidence for the presence of TDP-43 CTFs in both the ALS and FTLD brain, structural magnetic resonance imaging as well as post-mortem analysis reveals widespread brain atrophy in FTLD (Broe et al., 2003; Consonni et al., 2018; Sellami et al., 2018), whereas patients with ALS exhibit milder, focal thinning of the primary motor and pre-motor cortices and damage to the corticospinal tract (Roccatagliata et al., 2009; Chen and Ma, 2010; Verstraete et al., 2012). Further to this, TDP-43 CTFs are typically not detected in the ALS spinal cord (Neumann et al., 2006; Igaz et al., 2008; Uchida et al., 2012; Kwong et al., 2014), despite the dramatic tissue loss associated with disease progression (Branco et al., 2014). Instead, immunohistochemical studies have reported co-labeling of lower motor neuron inclusion bodies with antibodies directed against both the N- and C-termini of TDP-43, suggesting the inclusions primarily comprise the full-length protein (Igaz et al., 2008). The studies that have detected TDP-43 CTFs in the ALS spinal cord show that they are much less abundant than full-length TDP-43 (Neumann et al., 2009; Giordana et al., 2010; Smethurst et al., 2016). This indicates that TDP-43 CTFs may not be a prerequisite for neurodegeneration (Lee et al., 2011). The possibility that CTFs promote more rapid neurodegeneration and therefore fail to be detected in the ALS spinal cord at post-mortem seems unlikely, given that the fragments continue to be observed in the cortex regardless of cell loss. Further examination is therefore necessary to establish whether these fragments play a causal role in disease.

Numerous studies have attempted to correlate the relative abundance of the several TDP-43 CTFs between 18 and 27 kDa detected by immunoblotting with FTLD-TDP subtype or diagnosis of ALS. Despite progress in this area, low sample sizes, poor resolution of the immunoblots and heterogeneity in the neuropathology of patients with TDP-43 proteinopathies have obscured distinction of a reliable immunoblot pattern of CTFs (Arai et al., 2006, 2010; Hasegawa et al., 2008; Inukai et al., 2008). To address this, a large-scale biochemical examination of FTLD brain tissue from 42 individuals was conducted (Neumann et al., 2009). Although some similarity with previously reported patterns was observed (Hasegawa et al., 2008; Inukai et al., 2008), the overall relative abundance of low molecular weight species of TDP-43 could not be mapped to genetic risk factor or clinico-pathological subtype. A recent study reported that the levels of a 26 kDa CTF were higher than those of other lower molecular weight CTFs in FTLD-TDP patients with type E pathology compared to patients with types A, B, or C pathology (Lee E. B. et al., 2017). More sensitive methods of detection and quantification of various CTFs are required to fully elucidate their relative abundance in disease tissues.

## Generation of TDP-43 CTFs

In addition to transcriptional autoregulation, TDP-43 may be cleaved into smaller fragments before being enzymatically degraded to maintain physiological levels (Ling et al., 2010; Huang et al., 2014). TDP-43 is processed by a range of cysteine proteases, including caspases and calpains. In TDP-43 proteinopathies, disease-related factors such as cell stress and genetic mutations may modulate the activity of these enzymes. Assays of TDP-43 fragmentation using both mammalian cell lines and primary neuronal cultures frequently demonstrate the presence of CTF-35. This fragment is rarely observed in human post-mortem tissue (Neumann et al., 2006, 2009; Hasegawa et al., 2008) although interestingly, it has been reported in lymphoblastoid cells derived from ALS patients (Kabashi et al., 2008; Rutherford et al., 2008). Conversely, CTF-25, a pathological signature of the ALS and FTLD brain, was often undetected in these cell experiments, or was only observed at low levels. This brings into question the extent to which cell assays of TDP-43 cleavage can be extrapolated to human disease. It also suggests that the mechanisms of TDP-43 fragmentation and potentially the biochemical effects of the TDP-43 fragments may vary according to experimental model and biological sample (highlighted in Figure 1). These observations illuminate the importance of interpreting data from cell culture studies within the context of findings from human and animal investigations.

### Caspases

Prior to the discovery of TDP-43 as the primary component of insoluble inclusions in ALS and FTLD (Neumann et al., 2006), elevated caspase-3 activity had been noted in the anterior horn and motor cortex of ALS patients (Martin, 1999), as well as in degenerating astrocytes and neurons exhibiting DNA damage in the superior frontal gyrus and anterior pole of the temporal lobe of the FTLD brain (Su et al., 2000). Since then, numerous studies have confirmed TDP-43 as a caspase substrate (Thiede et al., 2005; Van Damme et al., 2005; Dix et al., 2008), with the TDP-43 amino acid sequence containing three putative caspase-3 consensus sites that correspond to CTFs of approximately 42, 35, and 25 kDa (Zhang et al., 2007). Experiments in human cell lines demonstrate cleavage of TDP-43 by a range of caspases including caspase-3, -4, and -7 (Rutherford et al., 2008; Cassel et al., 2012; De Marco et al., 2014; Li et al., 2015).

#### Cell Stress

Elevation of cleaved caspase-3 and -9 is associated with the generation of CTF-35 under conditions of cell stress. These include treatment with the classic apoptotic insult staurosporine (Dormann et al., 2009; Suzuki et al., 2011) or chemical inducers of endoplasmic reticulum (ER) stress such as thapsigargin, dithiothreitol, or tunicamycin (Suzuki et al., 2011), hyper-osmotic pressure induced by treatment with D-sorbitol (Dewey et al., 2011; Wobst et al., 2017), and chronic oxidative stress in the form of exposure to paraquat, an inhibitor of the mitochondrial electron transport chain (Meyerowitz et al., 2011). However, CTF-25 fragments were not observed in these studies (Meyerowitz et al., 2011; Wobst et al., 2017), or were only faintly detected (Dormann et al., 2009; Suzuki et al., 2011). In a rat model of acute stroke, ischaemic neurons demonstrated elevated levels of soluble CTF-25 and activated caspase-3 (Kanazawa et al., 2011).

Although numerous other studies have substantiated the effect of cellular stress on TDP-43 cleavage, they did not examine whether this occurred via caspase activation. For instance, arsenite-induced oxidative stress generated CTF-35, an effect which was dependent on increased translation of the stress response gene *GADD34* (Goh et al., 2018). Inhibition of the ubiquitin-proteasome system by treatment with epoxomicin or MG132 was associated with cytoplasmic TDP-43 fragments of approximately 32–35 kDa, and relatively low levels of CTF-25, in both immortalized neuronal cell lines and primary hippocampal and cortical cultures (Ayala V. et al., 2011; van Eersel et al., 2011). Proteasome inhibition also exacerbated the aggregation of ectopically expressed CTFs (Walker et al., 2013; Ishii et al., 2017). Interestingly, while decreased proteasomal activity has been reported in cell culture models of mutant *VCP*, which accounts for a small proportion of people with FTLD (Gitcho et al., 2009b), TDP-43 CTF-25 is variably detected at low levels in the brains of people with *VCP*-linked FTLD (Neumann et al., 2007).

#### Progranulin Deficiency

Mutations to the *GRN* gene have also been proposed to mediate caspase-induced cleavage of TDP-43, although the initial evidence for this has since been largely invalidated. The majority of *GRN* mutations linked to FTLD are non-sense or frameshift mutations resulting in haploinsufficiency (Lopez de Munain et al., 2008) or missense mutations affecting *GRN* stability (van der Zee et al., 2007), all of which ultimately confer a loss of function of the progranulin protein. An early study posited *GRN* deficiency as the molecular mechanism underlying TDP-43 cleavage by caspase-3, showing activation of caspase-3 and the formation of TDP-43 CTF-25 and CTF-35 following knockdown of the *GRN* transcript by RNA interference in HeLa and H4 neuroglioma cells (Zhang et al., 2007). However, this finding was not supported by subsequent experiments in multiple cell models. *GRN* knockdown in HeLa and SH-SY5Y neuroblastoma cells did not trigger caspase-3 activation or TDP-43 cleavage (Dormann et al., 2009). In another study, equivalent levels of TDP-43 fragments of 36 kDa and smaller were detected in the nuclei of HeLa cells transfected with *GRN* siRNA, as well as in control mock-transfected cells (Shankaran et al., 2008). This suggests that not all breakdown products of TDP-43 are associated with disease, mirroring other studies in which low levels of TDP-43 CTFs were detected under baseline conditions (Dormann et al., 2009; Caragounis et al., 2010; Nishimoto et al., 2010; Meyerowitz et al., 2011).

Animal models of *GRN* deficiency similarly fail to support a critical role for *GRN* in TDP-43 fragmentation. Mutant *GRN*^-/-^ mice do not exhibit TDP-43 truncation (Dormann et al., 2009) and display normal nuclear localization of TDP-43 and no difference in caspase-3 activation (Dormann et al., 2009; Ahmed et al., 2010). Caspase-3 activation and apoptosis were observed in mixed primary cortical cultures from *GRN*^-/-^ mice, however, no proteolytic processing of TDP-43 occurred (Kleinberger et al., 2010). Similarly, downregulation of *GRN* in zebrafish does not cause TDP-43 truncation or mislocalization (Shankaran et al., 2008). Although wild-type mice exhibit increased cytosolic TDP-43 and decreased progranulin levels in denervated motor neurons following sciatic axotomy, there is no evidence of TDP-43 fragmentation (Moisse et al., 2009).

#### The Physiological Relevance of Caspase Cleavage of TDP-43

Studies examining caspase-mediated cleavage of TDP-43 have primarily used cellular assays that rely on the induction of apoptosis, or examination of post-mortem tissue from end-stage ALS and FTD patients. As an executioner caspase that is typically activated during programmed cell death, it is plausible that caspase-3 activation is a late-stage event that occurs at the onset of apoptosis rather than driving the initial cellular dysfunction in TDP-43 proteinopathies. To investigate this possibility, neuronal cell lines were exposed to sub-lethal doses of inducers of oxidative or ER stress (hydrogen peroxide and thapsigargin, respectively) (Ayala V. et al., 2011). Importantly, increased levels of cleaved caspase-3 were not observed, indicating that the activation of caspase-3 and subsequent cleavage of TDP-43 occurs after other pathological events that cause the onset of cell dysfunction, and may be a relatively late event in TDP-43 pathology.

Alternatively, caspase-cleavage of TDP-43 may play a physiological role in the maintenance of TDP-43 homeostasis. Examination of the time-course of fragmentation following overexpression of full-length TDP-43 identified ER membrane-bound caspase-4 as being responsible for initial cleavage at asparagine residue 174 to produce CTF-25 (Li et al., 2015). This subsequently activated caspase-3/7 to accelerate the generation of a 35 kDa fragment. It was concluded that caspase-4 monitors the amount of full-length TDP-43 accumulated in the ER and cleaves a small portion of the protein when optimal levels are exceeded (Li et al., 2015). Interestingly, this caspase was also elevated in the brains of rhesus macaques injected with TDP-43 M337V, where TDP-43 CTF pathology was also observed (Yin et al., 2019). In another study, a TDP-43 mutant resistant to caspase cleavage triggered cell death more rapidly than wild-type TDP-43 (Suzuki et al., 2011). Based on these findings, caspase-cleavage may be a protective cellular response triggered by other pathological factors, such as excessive levels of full-length TDP-43.

### Calpains

The use of caspase inhibitors in studies of cell stress demonstrates that fragmentation of TDP-43 can occur via pathways that are independent of caspase processing (Meyerowitz et al., 2011; Nan et al., 2018). Calpains are a family of calcium-dependent, non-lysosomal cysteine proteases, the activation of which is modulated by the influx and efflux of Ca^2+^ into the cell (Baudry and Bi, 2016). Calpain activation may also contribute to TDP-43 truncation. This likely arises from dysregulation of Ca^2+^ and concomitant glutamate excitotoxicity, both of which have been broadly associated with pathological alterations in the brains of people with ALS and FTLD (Van Den Bosch et al., 2006; Murley and Rowe, 2018). Calpain has been identified as the major protease responsible for the cleavage of TDP-43 into CTF-25 and CTF-35 in two mouse models of traumatic brain injury, as well as cellular models of neurotoxicity (Yang et al., 2014). Other studies also point to a relationship between excitotoxicity and cleavage of TDP-43 but these did not examine the role of calpains. For instance, induction of excitotoxicity via blockade of glutamine transport produced a 37 kDa TDP-43 fragment in rat spinal cord organotypic slice cultures (Ayala V. et al., 2011). Further, TDP-43 was cleaved into CTF-25 and CTF-35 under conditions of restricted intracellular Ca^2+^ (De Marco et al., 2014). Although this was attributed to the activity of caspases rather than calpains, it nonetheless implicates general dysregulation of Ca^2+^ homeostasis in the cleavage of TDP-43 into CTFs.

Evidence from cell and *in vitro* assays has also linked calpain activation with the generation of fragments from the N-terminal portion of TDP-43, with a range of cleavage sites between residues 229–346 in the C-terminal domain (Yamashita et al., 2012). Enzyme levels of both calpain-1 and calpain-2 were elevated in the spinal cord and primary motor cortex of patients with FTLD and motor neuron disease (MND) where 25 and 35 kDa N-terminal fragments were detected (Yamashita et al., 2012). However, 35 kDa TDP-43 fragments are infrequently detected in human neuropathology studies, and the presence of N-terminal TDP-43 fragments has not been previously reported, despite systematic examination of ALS and FTLD patient tissue (Igaz et al., 2008). Therefore, the pathophysiological relevance of the 35 kDa N-terminal fragment remains to be determined.

### Alternatively Translated Isoforms

Several lines of evidence suggest that TDP-43 CTFs may also be produced via translation of an alternative transcript which is upregulated in disease. Site-specific mutagenesis and *in vitro* transcription/translation assays have identified two novel TDP-43 isoforms of 35 and 25 kDa which are generated under baseline conditions and in the absence of caspase activity (Nishimoto et al., 2010). The 35 kDa TDP-43 CTF may be accounted for by alternative in-frame translation from a downstream initiation codon of a specific splice variant which is upregulated in ALS spinal cords (Xiao et al., 2015). This variant has a 91 base pair splicing deletion at exon 2 and produces a gene product encoding a 35 kDa protein, proposed to be due to altered ribosomal scanning (Xiao et al., 2015). The presence of this protein was validated in ALS spinal cord tissue using an antibody directed against its predicted N-terminus, and when ectopically expressed in primary motor neurons it formed cytoplasmic aggregates and reduced cell viability (Xiao et al., 2015). In another study, analysis of alternatively spliced transcripts arising from the *TARDBP* locus in human cortex revealed three splice variants encoding a unique C-terminal sequence that directed localization of the expressed protein to the cytoplasm when transduced in human neuroglioma cells (D’Alton et al., 2015). However, the involvement of these proteins in human disease remains unclear, and there is as yet no evidence that the splice variants are expressed as proteins in the human brain. Nonetheless, although the generation of TDP-43 CTFs by post-translational modification to the TDP-43 protein is well established, it remains possible that CTFs may also arise from alterations at the transcriptional level.

### Effect of Disease-Linked Genetic Mutations on TDP-43 CTF Generation

Genetic mutations linked with TDP-43 proteinopathies and ALS/FTD, including mutations to *TARDBP*, *C9orf72*, ataxin 2 (*ATXN2*) and superoxide dismutase 1 (*SOD1*), may confer vulnerability to caspase-cleavage of TDP-43. Numerous *TARDBP* mutations are linked to both familial and sporadic ALS or FTD (Gitcho et al., 2008, 2009a; Kabashi et al., 2008; Sreedharan et al., 2008). The majority of these mutations reside within the C-terminal domain and it is therefore plausible that they affect the biochemistry of the TDP-43 C-terminal region, including its susceptibility to proteolytic cleavage (Pesiridis et al., 2009). In line with this, studies using lymphoblastoid cells from patients harboring different *TARDBP* mutations indicate that specific mutations may predispose TDP-43 to fragmentation under baseline conditions (Corrado et al., 2009) or during proteasomal inhibition (Kabashi et al., 2008; Rutherford et al., 2008). Similarly, TDP-43 CTFs were detected in the spinal cord of a patient with a mutation causing a Q343R amino acid substitution, whereas no CTFs were detected in other sporadic ALS cases (Yokoseki et al., 2008). Furthermore, the A315T mutation has been shown to form persistent TDP-43 fragments that are resistant to further degradation by proteases (Guo et al., 2011). The disease-associated D169G mutation, which resides in the RRM1 region outside the C-terminal domain (Kabashi et al., 2008), is also associated with caspase-3 cleavage due to the enhanced stability and hydrophobic interactions of the RRM1 core afforded by a subtle local conformational change to the ß-turn region containing the mutation (Chiang et al., 2016).

Hexanucleotide expansions in the *C9orf72* gene account for the largest proportion of familial ALS and FTLD cases (DeJesus-Hernandez et al., 2011; Renton et al., 2011; McCann et al., 2017). However, whether repeat-associated non-AUG (RAN) translation of the intronic GGGGCC hexanucleotide repeat in *C9orf72-*linked ALS and FTLD promotes TDP-43 fragmentation via caspase-3 activation has not been directly addressed. Overexpression of the glycine-alanine dipeptide repeat protein (poly GA), which is produced by RAN-translation of the *C9orf72* hexanucleotide expansion, in cultured cells induces caspase-3 activation and ER stress (Zhang et al., 2014), and poly GA peptides have also been associated with production of a TDP-43 fragment of approximately 30 kDa (Lee Y. B. et al., 2017). Further experiments addressing the precise link between *C9orf72* mutation and TDP-43 pathology, including proteolytic cleavage, are therefore warranted.

Elevation of activated caspase-3 has been noted in the spinal motor neurons of ALS patients harboring intermediate length polyQ expansions (27–33 CAG repeats) in *ATXN2*, which confers increased risk of ALS (Elden et al., 2010; Hart and Gitler, 2012). Expression of ataxin 2 with intermediate length glutamine tracts (31Q) in neuronal and non-neuronal cell lines increased the levels of a 30 kDa TDP-43 CTF (Hart and Gitler, 2012). Importantly, these effects on TDP-43 CTF generation were not observed when shorter (22Q, normal) or longer (39Q, spinocerebellar ataxia-associated) ataxin 2 proteins were expressed (Hart and Gitler, 2012). It is unclear whether *ATXN2* expansions are mechanistically linked to caspase activation and TDP-43 cleavage, although knockdown of endogenous ataxin 2 in transgenic TDP-43 mice ameliorates disease and may slightly decrease levels of insoluble TDP-43 CTF-35 (Becker et al., 2017).

Mutations to the SOD1 gene were the first reported cause of ALS (Rosen et al., 1993). Motor neuron-like NSC-34 cells form TDP-43 CTFs when incubated with aggregates of recombinant human SOD1 harboring the disease-causative G93A mutation (Zeineddine et al., 2017) or transfected with this particular mutant (Jeon et al., 2018). Some studies in SOD1 mutant mice (G85R and G93A substitutions) have also demonstrated the deposition of TDP-43 CTFs in the spinal cord, albeit at very low levels and late in disease (Cai et al., 2015; Jeon et al., 2018). However, another study found no evidence of TDP-43 fragmentation in SOD1 mice, and no mechanism underlying SOD1-mediated cleavage of TDP-43 has been proposed (Shan et al., 2009). Further to this, TDP-43 pathology is not found in neural tissue from people with ALS with SOD1 mutations (Mackenzie et al., 2007; Tan et al., 2007). It remains likely that any TDP-43 pathology associated with mutations to SOD1 is a minor player in disease and secondary to late-stage neurodegeneration.

## Degradation and Cellular Clearance of TDP-43 CTFs

In an effort to develop treatments to effectively clear TDP-43 pathology, numerous molecules have been identified as capable of degrading both full-length and fragmented TDP-43, including ubiquilin-2 (UBQLN2) (Cassel and Reitz, 2013) and poly(A)-binding protein nuclear 1 (PABPN1) (Chou et al., 2015). However, there are various conditions under which CTFs may be subjected to different clearance mechanisms compared to full-length TDP-43, which may contribute to their differential accumulation.

Molecular chaperones such as heat shock proteins (HSPs) are central to maintaining cellular proteostasis, including protein degradation via chaperone-mediated autophagy, and are commonly dysregulated in neurodegenerative diseases (Yerbury et al., 2016). The degradation of TDP-43 CTFs may be regulated by heat shock transcriptional factor 1 (HSF1) (Lin et al., 2016), which can induce HSP70. In line with this, the level of endogenous CTF-35 was unchanged following proteasome inhibition in cells overexpressing HSF-1 (Lin et al., 2016), which may be attributed to decreased caspase cleavage of TDP-43, or enhanced chaperone-mediated clearance of CTF-35. HSP70 levels were increased following expression of TDP-43 CTFs of varying sizes in cultured cells but this did not occur following full-length TDP-43 expression (Zhang et al., 2010). HSPB8 is another small HSP that may contribute to cellular clearance of TDP-43 fragments. In NSC-34 cells, upregulation of HSPB8 was greater following expression of CTF-25 and CTF-35 compared to full-length TDP-43, and the levels of insoluble CTF-25 were dramatically decreased following HSPB8 overexpression, compared to mild decreases in insoluble CTF-35 and full-length TDP-43 (Crippa et al., 2016).

The ubiquitin-proteasome system may also contribute to cellular clearance of TDP-43 CTFs. In cultured cells, the E3 ligase cullin-2 RING complex has been shown to enhance the ubiquitination and degradation of CTF-35 but not full-length TDP-43 (Uchida et al., 2016). Although it has been reported that the adaptor protein sequestresome 1 (p62/SQSTM1), which transports ubiquitinated proteins for proteasomal degradation, decreases aggregation of full-length TDP-43 as well as 15 and 25 kDa fragments (Brady et al., 2011), another study found p62 overexpression specifically lowered levels of CTF-35 but not full-length TDP-43 (Tanji et al., 2012). The proteasome has been shown to degrade a broad range of TDP-43 variants, including the full-length protein, disease-linked mutants and CTFs (Scotter et al., 2014; Chou et al., 2015; Wang X. et al., 2015; Crippa et al., 2016; Cicardi et al., 2018). However, CTF-25 is more sensitive to degradation by autophagy than CTF-35 or full-length TDP-43, as demonstrated by pharmacological inhibition or activation of the autophagy pathway (Scotter et al., 2014; Wang X. et al., 2015; Crippa et al., 2016; Cicardi et al., 2018). VCP has been shown to be important for functional autophagy (Ju et al., 2009), and mutations to *VCP* cause rare cases of FTLD-TDP type D. This suggests a potential link between VCP and the clearance of TDP-43. Expression of FTLD-linked VCP mutant proteins in cell culture increases cytoplasmic TDP-43 but does not lead to production of TDP-43 CTFs (Gitcho et al., 2009b). However, FTLD-TDP type D with *VCP* mutation is characterized by accumulation of intranuclear, not cytoplasmic, TDP-43 pathology. The cause of nuclear TDP-43 pathology in FTLD-TDP type D remains unclear, although the relatively low abundance of TDP-43 CTFs in these cases (Neumann et al., 2007) highlights the importance of extra-nuclear accumulation of TDP-43 in the generation of CTFs.

The N-terminal region of each TDP-43 CTF may determine the specific pathway through which it is degraded (Kasu et al., 2018). Arginyltransferase (ATE), the enzyme responsible for post-translational arginylation, has been implicated in signaling processes required for the degradation of intracellular proteins by autophagy or UPS pathways. Numerous proteins associated with neurodegenerative diseases have been identified as substrates of ATE, including β-amyloid, α-synuclein and TDP-43 CTFs (Galiano et al., 2016). The Arg/N-end rule pathway targets proteins and peptide fragments with destabilizing, unacetylated N-terminal residues, such as TDP-43 fragments cleaved at Arg208, Asp219, and Asp247, for proteolytic degradation (Brower et al., 2013). When expressed in mouse fibroblasts deficient for ATE, TDP-43 CTFs aggregate, suggesting that they are selectively degraded by the Arg/N-end rule pathway as a protective response to failing proteostasis and neurodegeneration (Brower et al., 2013). More recent work has shown that, whereas TDP-43 fragments beginning at residue 247 are degraded primarily by the Arg/N-end rule pathway, degradation of fragments with an N-terminal at residue 219 can occur independently of ATE (Kasu et al., 2018). Further dissection of the precise mechanisms of degradation of the different TDP-43 CTFs and the role of ATE is therefore warranted.

There are several experimental caveats to be considered when examining the degradation of TDP-43 CTFs. Several studies have demonstrated enhanced turnover of exogenously expressed TDP-43 CTFs compared to full-length TDP-43 or a TDP-43 protein containing a mutated NLS that drives cytoplasmic expression, henceforth referred to as TDP-43 ΔNLS (Pesiridis et al., 2011; Zhang et al., 2011; Hans et al., 2014; Huang et al., 2014; Scotter et al., 2014). Although CTFs are often more prone to aggregation than the full-length protein, soluble CTF-25 typically exhibits lower steady-state levels. Critically, this may be impacted by experimental conditions; these levels are elevated by tagging with eGFP compared to small tags such as HA or Myc (Li et al., 2011; Zhang et al., 2011; Scotter et al., 2014). Furthermore, whereas CTF-25 is often less soluble than other TDP-43 variants, assays of cleavage of endogenous TDP-43 demonstrate higher levels of CTF-35. It is possible that the cell lines used in these experiments are inherently more adept at recognizing and degrading CTF-35 or maintaining its solubility, which may underlie the high aggregation propensity of CTF-25 in these models.

## Expression of TDP-43 CTFs in Cell Models Variably Recapitulates Disease

In addition to the observation that neurodegeneration can proceed in TDP-43 proteinopathies in the absence of detectable TDP-43 CTFs, the argument for a disease-causing role for these fragments is weakened by the disparate results yielded by investigations into the biochemical effects of TDP-43 CTFs using *in vitro* and cell-based assays. Despite forming disease-reminiscent inclusions upon exogenous expression, TDP-43 CTFs typically do not confer a toxic gain of function, leaving markers of cytotoxicity and apoptosis unaltered. However, there is some evidence that they disrupt RNA splicing by TDP-43.

### Cytoplasmic Accumulation of Insoluble CTFs

TDP-43 CTFs accumulate in the cytoplasm of the cell, likely due to removal of the NLS during proteolytic cleavage (Winton et al., 2008; Lee et al., 2011). There is also evidence that CTFs form complexes with RNA transcripts to be transported out of the nucleus (Pesiridis et al., 2011). When expressed in cultured neuronal cells, fragments from the C-terminal portion of TDP-43 form ubiquitinated, phosphorylated inclusions that closely resemble human ALS and FTLD-TDP brain pathology (Igaz et al., 2009; Nonaka et al., 2009; Zhang et al., 2009, 2010; Yang et al., 2010; Fallini et al., 2012; Chou et al., 2015; Kitamura et al., 2016).

The propensity of TDP-43 to aggregate appears to be mediated by its C-terminal domain. Transduction of synthetic fibrils derived from short sequences within the TDP-43 C-terminal region has been shown to induce seed-dependent aggregation of full-length TDP-43 in neuroblastoma cells (Shimonaka et al., 2016). Deletion studies in yeast models determined that the presence of most of the TDP-43 C-terminal domain was necessary for aggregation to occur, and that aggregation increased according to how much of the RRM2 was included in the fragment (Johnson et al., 2008). Examination of recombinant TDP-43 *in vitro* revealed that amino acids 311–320 in the C-terminal domain were most critical for the formation of fibrils by TDP-43 CTFs (Saini and Chauhan, 2011). To complement these findings, removal of the glycine-rich domain (amino acids 265–319) in CTF-25 blocked heat shock-induced aggregation (Udan-Johns et al., 2014).

It is important to note that full-length TDP-43 is also inherently prone to aggregation and in the absence of mutations or modifications will spontaneously form aggregates that resemble those observed in ALS patients (Johnson et al., 2009). Moreover, neuroblastoma cells cultured with insoluble protein extracts from ALS brain tissue form aggregates of full-length TDP-43 prior to aggregates of CTFs (Nonaka et al., 2013). While aggregates of full-length TDP-43 are enriched for proteins involved in mRNA processing and RNA splicing, CTF-25 aggregates are enriched for proteins that moderate nucleocytoplasmic and intracellular transport (Chou et al., 2018), consistent with alterations to nuclear morphology observed following expression of this CTF (Walker et al., 2013). Thus, both full-length TDP-43 and its CTFs are prone to aggregation, and the aggregation of each may impact cellular functions via different mechanisms.

#### Factors That Modify TDP-43 CTF Aggregation

Cleavage of TDP-43 alone may not be sufficient to cause the insoluble inclusions that are characteristic of ALS and FTLD. Using an inducible form of the tobacco etch virus, it was shown that *de novo* cleavage of TDP-43 produced CTFs that were rapidly exported to the cytoplasm and cleared from the cell (Pesiridis et al., 2011). In this study, CTFs only formed inclusions when an additional insult occurred, such as microtubule transport disruption or depletion of RNA. RNA depletion has also been shown to exacerbate the sequestering of endogenous TDP-43 into CTF-25 aggregates (Kitamura et al., 2016). Phosphorylation may also modulate the solubility of TDP-43 CTFs. Indeed, the C-terminal domain of TDP-43 is enriched with serine residues, and phosphorylation of this region is widely observed in tissue from ALS and FTLD patients (Hasegawa et al., 2008; Inukai et al., 2008; Kametani et al., 2009; Neumann et al., 2009). Moreover, the majority of genetic mutations that map to the C-terminal domain of TDP-43 cause the abnormal creation or disturbance of serine or threonine residues, or the introduction of aspartic or glutamic acid phosphomimics (Buratti, 2018). TDP-43 CTFs transfected in immortalized cell lines were phosphorylated at the same or very similar sites to those seen in ALS and FTLD patient tissue and formed insoluble inclusions (Igaz et al., 2009; Zhang et al., 2010; Furukawa et al., 2011; Li et al., 2011). The effects of phosphorylation are likely dependent on the specific kinase involved and the site of phosphorylation.

Some studies suggest that phosphorylation has similar effects on both full-length TDP-43 and CTFs. For instance, inhibition of various kinase pathways, including c-Jun N-terminal kinase (JNK), cyclin-dependent kinase (CDK), and glycogen synthase kinase 3 (GSK3), attenuates the aggregation of full-length and truncated forms of TDP-43 (Meyerowitz et al., 2011; Moujalled et al., 2013). However, there is also evidence that the solubility of TDP-43 and its lower molecular weight species may be differentially impacted by phosphorylation. In line with a pathogenic effect for phosphorylation, the degree of phosphorylation and associated insolubility of fragments cleaved within the RRM2 domain of TDP-43 has been shown to be dependent on the precise cleavage site; phosphorylation and insolubility of the fragment increased as the cleavage site moved closer to the C-terminus (Furukawa et al., 2011). On the other hand, phosphorylation of TDP-43 CTFs may be a protective phenomenon. Substitution of S409/410 for an aspartic acid phosphomimic in 15 kDa CTFs increased their solubility and decreased aggregation (Brady et al., 2011). Importantly, this mutation had no effect on full-length TDP-43, illustrating that a specific post-translational modification can have distinct effects on different TDP-43 species.

#### Aggregation of TDP-43 CTFs Does Not Necessitate Cell Death or Dysfunction

Continued debate surrounds the role of protein aggregation in neurodegeneration. It has been suggested that the large inclusions that characterize ALS and FTLD neuropathology may be the product of an adaptive response aimed at protecting cells from more toxic oligomers or diffusible assemblies that may not be readily detectable by standard biochemical or microscopy techniques (Polymenidou and Cleveland, 2011). Recent research has demonstrated that smaller soluble species of mutant SOD1 and dementia-associated forms of tau are more toxic to cells than large, mature fibrils (Ghag et al., 2018; Zhu et al., 2018). It is possible that TDP-43 aggregation functions in a similar manner; soluble TDP-43 monomers and oligomers as well as micro- and macro-aggregates have been identified in cellular models of TDP-43 proteinopathy (Scotter et al., 2014), and in another study, preventing TDP-43 self-aggregation did not prevent cell death (Liu et al., 2013).

Although CTFs expressed in cell culture reliably form disease-like cytoplasmic inclusions, this does not always induce cellular toxicity or apoptosis. For instance, exogenous expression of full-length TDP-43 induced programmed cell death more readily than CTF-35, whereas CTFs of 25–27 kDa had no effect, despite their cytoplasmic aggregation (Suzuki et al., 2011; Yamashita et al., 2014). By contrast, one study showed that CTF-25 triggered a greater degree of ER-stress mediated apoptosis than CTF-35 or full-length TDP-43 (Wang X. et al., 2015). Evidence for the toxicity of CTFs relative to full-length TDP-43 expressed in neuronal cell lines is also variable. Elevated levels of lactate dehydrogenase (LDH), indicating cell death, have been reported in M17 neuroblastoma cells following CTF-25 expression (Zhang et al., 2009). However, another study found that although longer CTFs (from residues 86–414 or 170–414) impaired neurite outgrowth, they did not impact LDH release or activated caspase-3 levels in NSC-34 cells, despite this fragment being more aggregation prone than full-length TDP-43 (Yang et al., 2010).

Evidence from studies of endogenous TDP-43 CTFs also calls into question the relationship between CTF aggregation and disease. In experiments using inducible pluripotent stem cells derived from a patient carrying an ALS-linked M337V TDP-43 mutation, some clones exhibited higher levels of insoluble CTFs but this was not associated with apoptosis (Bilican et al., 2012). Thus, a critical finding from cell biology studies is that, regardless of cytoplasmic redistribution and aggregation propensity, TDP-43 CTFs do not reliably induce a toxic “gain of function” via cellular toxicity or activation of apoptotic pathways. Further, these studies highlight the need to assay factors additional to aggregation. For example, cellular atrophy, necrosis and the activation of cell stress responses may also contribute to the functional failure of specific neuronal populations in TDP-43 proteinopathies (Bodansky et al., 2010; Liachko et al., 2010; Matus et al., 2013).

#### The Role of Phase Transition in TDP-43 Accumulation

Emerging research into the effects of TDP-43 liquid–liquid phase separation on the dynamics of membraneless organelles such as stress granules offers an alternative way of viewing TDP-43 accumulation in ALS and FTLD. The role of the TDP-43 C-terminal domain in liquid–liquid phase separation has been well established (Dewey et al., 2011; Li et al., 2018a,b). However, recent research suggests that the N-terminal domain also contributes to this phenomenon (Wang A. et al., 2018; Wang L. et al., 2018). In cell culture, TDP-43 CTF-35 is recruited to stress granules when ectopically expressed (Nishimoto et al., 2010; Nihei et al., 2012) or when fragmentation is induced by chronic oxidative stress (Meyerowitz et al., 2011). However, CTF-25 formed fewer stress granules than CTF-35 or full-length TDP-43 (Liu-Yesucevitz et al., 2010). Stress granule markers have been shown to co-localize with TDP-43-positive inclusions in the ALS spinal cord (Liu-Yesucevitz et al., 2010; Bentmann et al., 2012), or partially colocalize with smaller punctate forms of TDP-43 (Colombrita et al., 2009). However, in the cortex of ALS and FTLD patients where CTFs predominate, stress granule markers do not co-localize with TDP-43 inclusions (Bentmann et al., 2012) or show a lower degree of specificity than in ALS spinal cord tissue (Liu-Yesucevitz et al., 2010). The fact that the RNA binding motifs of TDP-43 are intact in CTF-35 but not in CTF-25 (see Figure 1) (Wei et al., 2017) suggests that RNA-binding may be required for the formation of TDP-43-positive stress granules. Indeed, studies using deletion constructs have shown that both the RRM1 and C-terminal regions are necessary for the recruitment of TDP-43 to stress granules (Colombrita et al., 2009).

### Cell-to-Cell Propagation of TDP-43

The C-terminal region of TDP-43 shares moderate sequence homology with prion proteins (Guo et al., 2011). Given this structural similarity, a prion-like mechanism of self-templating propagation has been proposed to explain the focal onset and subsequent spread of dysfunction in ALS patients (Cushman et al., 2010; Polymenidou and Cleveland, 2011; Jucker and Walker, 2013; Ludolph and Brettschneider, 2015; Brauer et al., 2018). Consistent with this, mammalian cells incubated with protein or exosomes extracted from ALS and FTLD brain homogenates formed TDP-43 CTFs of the same molecular weight as those detected by immunoblotting of the brain lysates (Nonaka et al., 2009; Furukawa et al., 2011; Iguchi et al., 2016; Smethurst et al., 2016). In a similar manner, ALS spinal cord homogenates, which do not contain CTFs, induced the formation of aggregates of full-length TDP-43 in cell culture (Smethurst et al., 2016). The capacity to induce *de novo* pathology suggests that TDP-43 is capable of being transmitted to nearby or interconnected cells, where it may corrupt endogenous TDP-43 to adopt pathological, aggregation-prone conformations (Irwin et al., 2015). While the C-terminal domain has been postulated to mediate self-templating of TDP-43 (Mompean et al., 2016), the regional spreading of TDP-43 is not unique to CTFs, and the inoculation studies described above do not indicate exacerbated propagation of pathology by brain extracts containing CTFs compared to spinal cord extracts containing the full-length protein (Smethurst et al., 2016). Further, it has been proposed that the prion-like architecture of the C-terminal domain enables TDP-43 to form structurally flexible aggregates under physiological conditions (Wang et al., 2012). In this study, ALS-linked mutations to this region, such as G348C and R361S, decreased the prion-like activity of TDP-43, thereby triggering the formation of rigid pathological inclusions. Overall, despite the prion-like structure of the TDP-43 C-terminal domain, there is no evidence from cell assays that the templated inter-cellular transmission of TDP-43 pathology is impacted by the cleavage of TDP-43 into CTFs.

### Effects of CTFs on Endogenous Full-Length TDP-43

C-terminal fragments of approximately 30–35 kDa have been shown to sequester endogenous TDP-43 from the nucleus, resembling the clearance of nuclear TDP-43 seen in people with ALS and FTLD-TDP (Nonaka et al., 2009; Nishimoto et al., 2010; Che et al., 2011, 2015). On the other hand, co-immunoprecipitation experiments have demonstrated that TDP-43 CTF-25 binds weakly to native full-length TDP-43 (Nonaka et al., 2009; Zhang et al., 2009). Although this resulted in depletion of nuclear TDP-43 in one study (Nonaka et al., 2009), another study reported no sequestration of endogenous TDP-43 (Zhang et al., 2009).

### CTFs May Impair RNA Processing by TDP-43

Evidence for perturbation of RNA metabolism following cleavage of TDP-43 is more consistent. Impairment of skipping of exon 9 of the cystic fibrosis transmembrane conductance regulator (CFTR) pre-mRNA, used as a model of TDP-43 function, has been observed for both CTF-25 (Igaz et al., 2009; Nonaka et al., 2009; Zhang et al., 2009; Nishimoto et al., 2010; Udan-Johns et al., 2014) and CTF-35 (Nishimoto et al., 2010; Che et al., 2011, 2015). However, one study reported no effect on exon skipping following expression of a CTF from residues 220–414 in neuroblastoma cells, corresponding to the caspase-cleaved CTF-25 identified in human disease (Zhang et al., 2009). This may be because the glycine/asparagine-rich region of the TDP-43 C-terminal domain contributes to exon skipping activity and other aspects of gene transcription (Wang et al., 2004, 2012; Buratti et al., 2005). As a result, partial or total removal of the RNA recognition motifs of TDP-43 following cleavage of its N-terminal domain might not completely disrupt RNA processing. Thus, cell-based examinations suggest that TDP-43 CTFs may contribute to cellular dysfunction via a loss of normal function, impairing the ability of TDP-43 to regulate RNA splicing.

## TDP-43 CTF Expression *IN VIVO* Poorly Models ALS and FTLD Phenotypes

In both ALS and FTLD, TDP-43 pathology is observed in a range of cell types and may spread from a focal point of onset to affect other regions of the brain and spinal cord (Ravits and La Spada, 2009; Brettschneider et al., 2013, 2014). A key limitation of cellular models of ALS and FTLD is that they do not recapitulate the complex anatomy involved in TDP-43-driven neurodegeneration (Van Damme et al., 2017). For this reason, it is imperative that the validity of *in vitro* findings is confirmed in appropriate animal models. While simple model organisms such as *Drosophila melanogaster*, *Caenorhabditis elegans* and zebrafish are easily manipulated for microscopy and offer well-characterized genomes, rodent models more faithfully reproduce the cellular and behavioral or motor phenotypes that typify TDP-43 proteinopathies (Dayton et al., 2013). Accordingly, rodent models are particularly useful for illuminating disease mechanisms and for pre-clinical testing of potential therapeutics (Van Damme et al., 2017), and are the most broadly employed *in vivo* models of ALS and FTD (Tan et al., 2017a).

In order to examine the contribution of truncated forms of TDP-43 to motor or cognitive decline in ALS and FTD, several transgenic rodent models expressing TDP-43 CTFs of approximately 25 kDa have been developed. These models have typically used broad neuronal promoters such as *Thy1.2*, mouse prion protein (*PrP*) and neurofilament heavy chain (*NEFH*) to direct selective transgene expression in neurons of the brain and spinal cord. Some models have employed the Ca^2+^/calmodulin-dependent kinase II α (*CaMKII*α) promoter to restrict expression to forebrain neurons, primarily in the cortex, hippocampus, and striatum (Cannon et al., 2012), making them particularly relevant for the investigation of FTLD. When modeling ALS and FTLD, the choice of gene promoter is pertinent as CTFs are rarely detected in the spinal cord of patients, regardless of which disease is manifested.

### Transgenic TDP-43 CTF Expression Modestly Affects Motor Performance and Cognition

Numerous animal models have considered the role of TDP-43 CTF-25 in the onset and progression of ALS and FTD, summarized in Table 2. Remarkably, transgenic overexpression of TDP-43 CTFs in behaving animals consistently fails to recapitulate the key behavioral features of these diseases. Moreover, the neuropathology observed following CTF expression in animals is incongruent with findings in cell and human post-mortem studies. These animal models therefore provide the most compelling evidence that TDP-43 CTFs alone are unlikely to be prime triggers of neurodegenerative disease.

**Table 2:** Few overt effects on neuropathology and motor and behavioral phenotypes are seen in overexpression studies of TDP-43 CTFs in mouse, rat, *Drosophila*, and *C. elegans* models.

Model organism	Amino acid sequence of TDP-43 CTF transgene	Promoter/driver	Additional study details	Controls and comparisons	Phenotype measure	Phenotype outcome	Neuropathology	References
Mouse	208–414	*CaMKII*α	Dox-suppressible expression. Transgene induced at 1 month of age. Mice examined up to 24 months of age.	NT and monogenic littermates	Rotarod Wirehang Y-maze	No gross motor or cognitive deficits.	CTFs localized to cytoplasm but no large inclusions. CTFs phosphorylated in hippocampal CA1 region but no neurodegeneration. Minimal phosphorylation of CTFs in the dentate gyrus but progressive death of granule cells in mice 10 months and older.	Walker et al., 2015b
			Insoluble protein extracted from FTLD- TDP brains injected into mouse cortex.	NT and ΔNLS mice	n/a		Mice demonstrated less spread of TDP-43 pathology throughout the brain compared to ΔNLS mice.	Porta et al., 2018
		*NEFH*	Dox-suppressible expression. Transgene induced at 1 month of age. Mice examined up to 19 months of age.	NT and monogenic littermates	n/a		No overt neurodegeneration. TDP-43 phosphorylated in CA1 region of hippocampus, and ubiquitinated in SLM.	Walker et al., 2015b
			Dox-suppressible expression. Transgene induced for 6 weeks in adult mice.	NT	No gross motor defects observed (data not shown).		No motor neuron degeneration, clearance of nuclear TDP-43 or large cytoplasmic inclusions detected in lumbar SC.	Spiller et al., 2018
		*PrP*	Animals examined at 8, 13, and 18 months of age.	FL-TDP-43 and NT	Rotarod Y-maze Contextual and cued fear conditioning	No motor or behavioral phenotype. Mild memory and motor deficits were observed in mice that overexpressed FL-TDP-43.	No change in endogenous TDP-43 levels.	Tsuiji et al., 2017
	216–414	*Thy1*.*2*	Animals examined at 2 or 6 months of age.	NT littermates	Novel object recognition test T-maze Open field locomotion test Rotarod	No significant difference in any behavior or motor tests.	Soluble CTFs detected in cytoplasm and nucleus, by long exposure of immunoblot. No change in endogenous TDP-43 levels and no inclusions or neurodegeneration.	Caccamo et al., 2012
			Mice treated with the glucocorticoid dexamethasone at 6 months of age.	NT	Spatial version of Morris water maze	Dexamethasone treatment worsened memory deficits but had no effect on swim speed or distance traveled, indicating no motor impairment.	In mice expressing CTF-25, dexamethasone treatment impaired autophagy (indicated by lower levels of Atg7 and LC3-II) and increased soluble CTF-25 levels in the nucleus and cytoplasm but no change to endogenous FL-TDP-43 levels.	Caccamo et al., 2013
			Hemizygous and homozygous mice examined at 15 months of age.	NT	Radial arm water Maze Morris water maze Rotarod	Hemizygous mice exhibited motor dysfunction and impaired spatial and working memory, exacerbated in homozygous mice.	Impaired autophagy and proteosomal function in both lines. Soluble and insoluble CTF-25 detected in the nucleus and cytoplasm, but FL-TDP-43 only detected in the nucleus. Homozygous mice showed lower levels of endogenous FL TDP-43.	Caccamo et al., 2015
	219–414	CMV/chicken β-actin	*In utero* electroporation of construct into embryonic motor cortex.	FL-TDP-43 and TDP-43^M337V^	n/a		CTFs formed ubiquitinated, phosphorylated aggregates in nucleus and cytoplasm, detected up to 21 days post-natal.	Akamatsu et al., 2013
Rat	220–414	CMV/chicken β-actin	IV injection of AAV9 in WT neonatal rats. Animals phenotyped from 2 to 24 weeks of age.	ΔNLS and GFP	Rotarod Open field test Hindlimb extension Locomotor Scoring of rearing behavior	CTF and ΔNLS rats showed equivalent motor dysfunction in rotarod and open field tests. Deficient forelimb use during rearing in seven of 13 CTF and two of 16 ΔNLS rats tested.	No neurodegeneration or muscle atrophy in any of the models examined.	Dayton et al., 2013
*Drosophila*	174–414 and 206–414	*elav*/*Gal4* for pan-neuronal expression, *D42*/*Gal4* for MN expression, *DaG32*/*Gal4* for ubiquitous expression		FL-TDP-43, ΔNLS, and disease-linked mutants (TDP-43^A315T^, TDP-43^G287S^, TDP-43^A382T^, and TDP-43^N390D^)	Climbing assay Longevity assay	Pan-neuronal or MN specific CTF expression caused a milder reduction in locomotion and lifespan compared to ΔNLS and disease-linked mutants. CTF had no impact on lifespan while other mutants caused premature death.	n/a	Voigt et al., 2010
	202–414	*GMR-Gal4* for expression in retinal photoreceptors		FL-TDP-43 and RFP	n/a		FL-TDP-43 associated with progressive degeneration and ultrastructural vacuoles in retinal cells, while CTF expression had no effect.	Li et al., 2010
				NT and FL-TDP-43	Climbing assay Longevity assay	Compared to FL-TDP-43, expression of CTF caused later onset of motor deficits, which was rescued by treatment with the small heat shock protein CG14207.	CTF-25 was less toxic than FL-TDP-43, produced a milder rough eye phenotype and was cleared more efficiently by CG14207.	Gregory et al., 2012
		*OK107-Gal4* for expression in mushroom bodies		FL-TDP-43 and RFP	n/a		FL-TDP-43 was associated with neuronal death and loss of axons. No effect of CTF expression.	Li et al., 2010
		*OK371-Gal4* for MN expression		FL-TDP-43 and RFP	n/a		CTF expression had no effect. Regions where FL-TDP-43 had accumulated in the cytoplasm showed axonal swelling, inclusion formation in soma and axons and fragmentation of the nucleus.	Li et al., 2010
*C. elegans*	219–411	*snb-1* for pan-neuronal expression		FL-TDP-43, disease-linked TDP-43^Q331K^ and TDP-43^M337V^ mutants	Fly motility assay	FL-TDP-43 and disease mutants produced more severe motor deficits than expression of the CTF.	CTF formed cytoplasmic aggregates but less toxic to neurons than FL-TDP-43.	Zhang et al., 2011
	220–411	*snb-1*		Constructs with RMM1, RMM2 or C-terminal deleted	n/a		CTF formed large cytoplasmic inclusions whereas RMM1 and RMM2 mutants had a granular appearance and C-terminal deletion mutant formed a singular aggregate in the cytoplasm.	Ash et al., 2010


*AAV9, adeno-associated virus serotype 9; Atg7, autophagy-related protein 7; *C. elegans*, *Caenorhabditis elegans*; CA1, cornu ammonis field 1; CaMKIIα, Ca^2+^/calmodulin-dependent kinase II α; CMV, cytomegalovirus; CTF; C-terminal fragment; Dox, doxycycline; FL-TDP-43, full-length TDP-43; GFP, green fluorescent protein; IV, intravenous; LC3, microtubule-associated protein 1A/1B-light chain 3; LC3-II, LC3-phosphatidylethanolamine conjugate; MN, motor neuron; NT, non-transgenic; RFP, red fluorescent protein; RRM1, RNA recognition motif 1; RRM2, RNA recognition motif 2; SC, spinal cord; SLM, stratum lacunosum-moleculare; snb-1, synaptobrevin-1; WT, wild-type.*

In transgenic mice, using the *CaMKII*α promoter for forebrain expression or the *NEFH* promoter to direct expression of CTF-25 (spanning amino acids 208–414) to the neurons of the brain and spinal cord does not induce motor or cognitive defects (Walker et al., 2015b; Spiller et al., 2018). The same CTF has been expressed in mice using the *PrP* promoter (for brain and spinal cord distribution) and again, no behavioral phenotype was reported (Tsuiji et al., 2017). By contrast, pan-neuronal expression of TDP-43 ΔNLS, for example, generates a dramatic and progressive motor phenotype akin to ALS (Walker et al., 2015a). Therefore, the inability of TDP-43 CTF-25 to induce behavioral alterations in mice cannot be explained by the choice of promoter.

In a different mouse model, CTF-25 (TDP-43 amino acids 216–414) was expressed in neurons using a *Thy1.2* promoter system (Caccamo et al., 2010, 2012, 2013, 2015). However, transgenic animals did not display any significant disease phenotype at 2 or 6 months of age on tests of motor function or measures of behavioral changes associated with FTD, such as the novel object recognition test (Caccamo et al., 2012, 2013). Performance of these animals on tests of working and spatial memory, such as the radial arm water maze and the Morris water maze, indicated memory impairments under specific conditions, such as aging and stress hormone modulation (Caccamo et al., 2013, 2015). However, changes to memory are rarely detected in the early stages of FTD (Bang et al., 2015). Hippocampal-dependent tasks, such as those used in these studies, are more appropriate measures of Alzheimer’s disease and dementia subtypes characterized by memory loss (Roberson, 2012; Vernay et al., 2016). Consistent with the memory impairments observed in these mouse models, TDP-43 CTFs arising from caspase cleavage at residue 219 have been detected in neurofibrillary tangles, Hirano bodies and reactive astrocytes in the hippocampus and entorhinal cortex of people with Alzheimer’s disease at post-mortem (Rohn, 2008). At 15 months of age, but not at earlier timepoints, *Thy1.2* CTF-25 mice performed significantly worse on rotarod test of motor co-ordination but showed no difference in swim speed in the Morris water maze (Caccamo et al., 2015). Overall, these studies indicate that under certain conditions, CTF-25 may cause some neuronal dysfunction, but this is not dramatic and is not highly reminiscent of ALS or FTD.

A rat model of TDP-43 CTF-25 (amino acids 220–414) has been produced using a viral-mediated strategy, resulting in highest expression in the spinal cord, cerebellum, muscle, heart, and liver (Dayton et al., 2013). Approximately half of the animals tested displayed mild forelimb impairment but exhibited no evidence of neuron loss or muscle wasting, in stark contrast to the widespread muscle atrophy and rapidly progressing motor dysfunction that is characteristic of people with ALS. It is also unclear how off-target effects due to heavy expression of the transgene outside the central nervous system (CNS) may have contributed to the results obtained. With regard to human pathology, it is critical to note that CTFs are rarely detected in the post-mortem ALS spinal cord and have not been reported in muscle (Weihl et al., 2008; Cykowski et al., 2018) or other peripheral tissues. The relevance of TDP-43 CTF expression outside the brain to the study of TDP-43 proteinopathies is therefore debateable.

Data from simple model organisms similarly suggest that TDP-43 CTFs are not key drivers of disease. In *Drosophila*, transgenic expression of full-length TDP-43 or ALS/FTLD-linked mutations has been shown to shorten overall lifespan (Voigt et al., 2010) and exacerbate age-dependent reductions in fly motility (Li et al., 2010) more dramatically than expression of TDP-43 CTFs. Overall, transgenic expression of TDP-43 CTFs in a range of model organisms does not induce dramatic motor or behavioral alterations that resemble TDP-43 proteinopathies.

### Neuropathology of CTFs in Animal Models Differs From Findings in Cell Culture

In addition to the poor recapitulation of disease-relevant behavioral and cognitive phenotypes in animal models of TDP-43 CTFs, the neuropathology in these animals also largely fails to capture that found in human ALS and FTD tissues. Perplexingly, the lack of clear TDP-43 pathology in animal models of TDP-43 CTFs differs from the consistent formation of TDP-43 inclusions that occur upon CTF overexpression in cell lines. Thus, data from animal models suggest that CTF expression alone is not sufficient to cause TDP-43 pathology *in vivo*, even when expression levels are several times greater than those of endogenous full-length TDP-43.

#### TDP-43 CTFs Rarely Form Inclusions in Animal Models

In contrast to the disease-reminiscent inclusions of TDP-43 CTFs observed in cell culture models, transgenic mice expressing TDP-43 CTFs exhibit microscopically diffuse CTFs with no evidence of large inclusions (Caccamo et al., 2012; Walker et al., 2015b; Spiller et al., 2018). In the human brain, antibodies against phosphorylated TDP-43 typically demonstrate high specificity when labeling TDP-43 macro-aggregates. However, in the mouse brain these antibodies indicate phosphorylation of diffuse CTF-25 (208–414). This occurs primarily in the CA1 region of the hippocampus despite expression of CTF-25 throughout the entire forebrain brain (Walker et al., 2015b). Likewise, phosphorylated but dispersed CTF-25 (220–414) has been detected in spinal cord motor neurons in virally transduced rats, without the presence of inclusions (Dayton et al., 2013), while *in utero* electroporation of CTF-25 (219–414) into the mouse motor cortex resulted in phosphorylated micro-aggregates detectable up to 3 weeks of age (Akamatsu et al., 2013). Although this discrepancy between inclusion formation and TDP-43 phosphorylation requires further study, these findings indicate that phosphorylation of TDP-43 CTFs can proceed even when the protein is diffusely distributed and may not necessarily result in the formation of large inclusions that are reflective of TDP-43 proteinopathies.

Studies using TDP-43 CTF mice have yielded disparate results as to the solubility of TDP-43 CTFs. Immunoblotting of mouse brains expressing CTF-25 (216–414) detected the fragments as detergent-soluble in both nuclear and cytosolic fractions (Caccamo et al., 2012, 2013, 2015), whereas transgenic expression of CTF-25 (208–414) caused accumulation of the CTFs almost exclusively in the detergent-insoluble fraction of the mouse brain (Walker et al., 2015b), in line with human neuropathology studies (Neumann et al., 2006; Igaz et al., 2008). In *C. elegans*, ectopically expressed CTF-25 (219–411 or 220–411) forms cytoplasmic aggregates (Ash et al., 2010; Zhang et al., 2011). Importantly however, exogenous full-length TDP-43 was shown to be more toxic than CTF-25 (Zhang et al., 2011). Together, these studies demonstrate that in complex tissues such as the brain and spinal cord, expression of TDP-43 CTFs does not reliably induce disease-reminiscent inclusion morphology.

#### TDP-43 CTFs Cause No or Minimal Neurodegeneration in Animal Models

Converging evidence from various model organisms substantiates the argument that expression of TDP-43 CTFs does not effectively replicate the progressive neuronal dysfunction and degeneration that is observed in people with TDP-43 proteinopathies. Rodent models of TDP-43 CTFs generally do not exhibit overt neurodegeneration or cell death in relevant neuronal populations (Caccamo et al., 2012; Dayton et al., 2013; Spiller et al., 2018). Consistent with this, studies in *Drosophila* have repeatedly demonstrated that full-length or disease-associated TDP-43 mutants are more toxic to retinal and neuronal cells than CTFs (Li et al., 2010; Voigt et al., 2010; Gregory et al., 2012). In CTF-25 (208–414) mice that did show neuron loss, this occurred specifically in the dentate gyrus of the hippocampus in the absence of TDP-43 inclusions and was not found in the CA1 region that contained most of the phosphorylated CTF-25 (Walker et al., 2015b). This indicates that even if neurodegeneration occurs following TDP-43 CTF expression in rodents, it is not associated with key features of human ALS and FTLD-TDP neuropathology. While post-mortem analysis of neural tissue from people with TDP-43 proteinopathies demonstrates the sequestering of TDP-43 to the cytoplasm (Neumann et al., 2006), TDP-43 CTF expression does not impact the levels or localization of endogenous nuclear TDP-43 in mice (Walker et al., 2015b; Tsuiji et al., 2017). The lack of fundamental neuropathology signatures of TDP-43 proteinopathies following expression of TDP-43 CTFs in animals suggests that rather than being a central driver of disease, CTFs are instead a by-product or consequence of mechanisms that do not directly cause neurodegeneration.

#### TDP-43 CTFs Promote Cell-to-Cell Transmission of TDP-43 Pathology Less Readily Than Full-Length Cytoplasmic TDP-43

The propagation of pathogenic TDP-43 to interconnected cells has long been hypothesized based on staged immunohistochemical experiments in post-mortem tissue (Brettschneider et al., 2013, 2014), and has been demonstrated in cell culture (Furukawa et al., 2011; Nonaka et al., 2013; Smethurst et al., 2016). However, this phenomenon was only recently confirmed in behaving animals (Porta et al., 2018). In this seminal study, delivery of insoluble extracts from FTLD-TDP brains into the cerebral cortex of mice initiated the progressive spread of TDP-43 inclusion pathology to anatomically connected regions. Examination of this event in several transgenic mouse models revealed that TDP-43 CTFs provide a cellular environment that is less conducive to the self-templated aggregation of TDP-43; injected brain extracts seeded *de novo* pathology more rapidly and widely in mice expressing TDP-43 ΔNLS than in mice expressing a TDP-43 CTF (208–414) (Porta et al., 2018). TDP-43 CTFs do not therefore appear to be primarily responsible for the propagation of TDP-43 pathology throughout the CNS in ALS and FTLD.

### The Presence of TDP-43 CTFs in Other Animal Models of ALS and FTLD Establishes TDP-43 CTFs as Disease-Associated Rather Than Disease-Causative

In Table 3, we present a comprehensive overview of the presence of TDP-43 CTFs in other animal models of TDP-43 proteinopathy which do not express TDP-43 CTFs alone. These models generally capture aspects of motor or behavioral dysfunction associated with ALS and FTLD more reliably than models of CTF overexpression, demonstrating more dramatic phenotypes and many of the neuropathological hallmarks of these diseases. Low levels of CTF-35, and even lower levels of CTF-25, are frequently observed in the brain and/or spinal cord of mice overexpressing wild-type TDP-43, or disease-associated mutations such as A315T, M337V and G348C amino acid substitutions (Wegorzewska et al., 2009; Stallings et al., 2010; Tsai et al., 2010; Wils et al., 2010; Xu et al., 2010, 2011; Swarup et al., 2011; Cannon et al., 2012; Janssens et al., 2013; D’Alton et al., 2014; Ke et al., 2015). In some of these models, TDP-43 CTFs are often difficult to detect by standard immunoblotting (Wegorzewska et al., 2009; Xu et al., 2011).

**Table 3:** Summary of studies examining the presence of TDP-43 CTF-25 and CTF-35 in transgenic mouse models of ALS/FTLD expressing wild-type or mutant forms of TDP-43.

Transgene	Promoter	Tissue analyzed	CTF-25	CTF-35	Other CTFs	Major findings	Reference
Wild-type hTDP-43	*PrP*	SC	+	+	-	Low levels of CTFs. Stronger labeling of FL-TDP-43 in nucleus and cytoplasm.	Stallings et al., 2010
		SC	+	+	-	Levels of FL-TDP-43 and CTFs was dose-dependent, with highest levels in homozygous mice.	Xu et al., 2010
	*CaMKII*α	Brain	+	+	-	An antibody against total TDP-43 detected low levels of fragments in NT animals, and higher abundance in transgenic animals.	Cannon et al., 2012
		Brain	-	-	-	No CTFs detected despite progressive motor dysfunction.	Igaz et al., 2011
	*Thy1.2*	Brain	+	++	-	CTF-35 present from disease onset, CTF-25 increased by end stage.	Janssens et al., 2013
		Brain	+	++	-	Levels of soluble CTF-25 increased as disease progressed, whereas CTF-35 levels decreased. CTF-35 cytoplasmic, CTF-25 cytoplasmic and nuclear.	Wils et al., 2010
	*Tardbp*	Brain and SC	-	-	-	Very low or no CTF-35 and no CTF-25.	Swarup et al., 2011
Wild-type mTDP-43	*CaMKII*α	Brain	+	++	-	Very low levels of fragments in transgenic animals aged to 6 months, but not animals aged to 2 months.	Tsai et al., 2010
hTDP-43^A315T^		Brain	-	+	15 and 20 kDa	CTFs less abundant than FL-TDP-43. Insoluble fragments highly phosphorylated at serine 409/410, while only some phosphorylation of the FL-TDP-43 was detected.	Ke et al., 2015
	*PrP*	Brain	+	+	-	CTF-25 detected in cytosol of mutant mice and NT controls. CTF-35 detected in nucleus and cytosol of transgenic animals only. CTFs less abundant than FL-TDP-43.	Medina et al., 2014
		SC	+	+	-	CTFs present in cytosol. FL-TDP-43 detected in nuclear and cytosolic fractions, and more abundant than CTFs.	Stallings et al., 2010
		Brain and SC	+	++	-	Low level detection of soluble CTFs prior to symptom onset and as disease progressed.	Wegorzewska et al., 2009
	*Tardbp*	Brain and SC	+	++	-	Higher levels of CTFs in mice aged to 10 months, but FL-TDP-43 more abundant overall.	Swarup et al., 2011
hTDP-43^M337V^	*PrP*	SC	+	+	-	Multiple faint bands between 25 and 35 kDa in cytosol, overall higher levels of FL-TDP-43 in nuclear and cytosolic fractions.	Stallings et al., 2010
		Brain	+	++	-	CTFs detected in both transgenic and NT mice by long exposure of immunoblot. FL-TDP-43 more abundant than CTFs.	Xu et al., 2011
	*Thy1.2*	Brain	+	++	-	CTF-35 present from disease onset, CTF-25 increased at end stage. Both lower than FL-TDP-43.	Janssens et al., 2013
	*CaMKII*α	Cortex	+	++	-	Fragments were detected by antibodies against RRM2 of TDP-43 but not amino acids 3–12 or 404–414.	D’Alton et al., 2014
	*TARDBP*	Brain and SC	-	+	-	CTF-35 detected in cortex of transgenic and NT animals but not in SC.	Gordon et al., 2019
hTDP-43^G348C^	*Tardbp*	Brain and SC	+	+	-	Higher levels of CTFs in mice aged to 10 months, but CTFs overall less abundant than FL-TDP-43.	Swarup et al., 2011
ΔNLS	*NEFH*	SC	-	+	-	Very low levels of CTF-35 in both transgenic and control animals.	Walker et al., 2015a
	*CaMKII*α	Brain	-	-	-	No detection of CTFs.	Igaz et al., 2011


*“+” indicates that the fragment was present, “-” indicates that it was not detected, and “++” indicates higher abundance of one fragment relative to the other. CA3, cornu ammonis field 3; CTF, C-terminal fragment; CTF-25, TDP-43 CTF of 25 kDa; CTF-35, TDP-43 CTF of 35 kDa; Dox, doxycycline; FL-TDP-43, full-length TDP-43; h TDP-43, human TDP-43; NT, non-transgenic; RRM2, RNA recognition motif 2; SC, spinal cord.*

Numerous attempts have been made to correlate the abundance of TDP-43 CTFs with disease stage in animal models, in order to disentangle the functional relevance of CTFs to neurodegeneration. A study by Wegorzewska et al. (2009) provides the only compelling evidence that CTFs may contribute to the initiation of disease, demonstrating the presence of CTFs prior to symptom onset in TDP-43^A315T^ mice. However, this conflicts with studies in various other model organisms which found that CTFs were only detectable at later disease stages. These findings support the argument that CTFs accumulate as a consequence rather than cause of neurodegeneration (Zhou et al., 2010; Uchida et al., 2012). For instance, CTFs accumulated in the mouse brain and/or spinal cord as symptoms of motor dysfunction progressed (Wils et al., 2010; Swarup et al., 2011; Janssens et al., 2013). In a rat model, insoluble CTFs were detected in animals that had reached paralysis, but were not detected at disease onset (Zhou et al., 2010). In a cynomolgus monkey, viral mediated delivery of wild-type TDP-43 to the cervical spinal cord induced motor dysfunction and muscle atrophy and, although low levels of CTF-25 was observed 4 weeks after injection, it was not present in early or mid-stage disease (Uchida et al., 2012).

Mice expressing TDP-43 ΔNLS show no or minimal TDP-43 fragmentation, despite a robust motor phenotype (Igaz et al., 2011; Walker et al., 2015a). The same observations have been made in mice that express ALS-linked TDP-43 mutations to levels equivalent to endogenous TDP-43, diminishing the status of CTFs as either disease-causing or disease-associated (Arnold et al., 2013). Moreover, some experiments have detected equivalent levels of CTFs in both transgenic animals and non-transgenic controls (Xu et al., 2011; D’Alton et al., 2014; Medina et al., 2014; Gordon et al., 2019). These studies provide convincing evidence that CTFs are not necessary to induce neurodegeneration, even in models that successfully phenocopy multiple aspects of human disease. However, it is interesting to note that although CTFs are present in low levels in both the brain and spinal cord of mice, transgenic expression of TDP-43 in pigs was associated with more prominent fragment pathology in the brain compared to the spinal cord (Wang G. et al., 2015), similar to the distribution seen in humans. In line with this, CTFs were more abundant than full length TDP-43 in M337V transgenic macaques, whereas mice expressing the same transgene showed lower relative abundance of fragments (Yin et al., 2019). This was replicated *in vitro*; recombinant TDP-43 M337V was cleaved into C-terminal fragments when incubated with monkey but not mouse brain extracts. This points to potential species-specific differences in the way that TDP-43 is processed. Indeed, the *TARDBP* gene of mammals is highly homologous with that of *Drosophila* and *C. elegans* from the N-terminal to RRM2, and diverges toward the C-terminus (Wang et al., 2004). Closer investigation is required to determine any differences in the C-terminal structure of primate and rodent TDP-43, and whether species differences affect TDP-43 processing.

## Cell-Type-Specific Differences in the Generation and Clearance of TDP-43 CTFs: a Potential Explanation for Regional Heterogeneity

**FIGURE 2:**
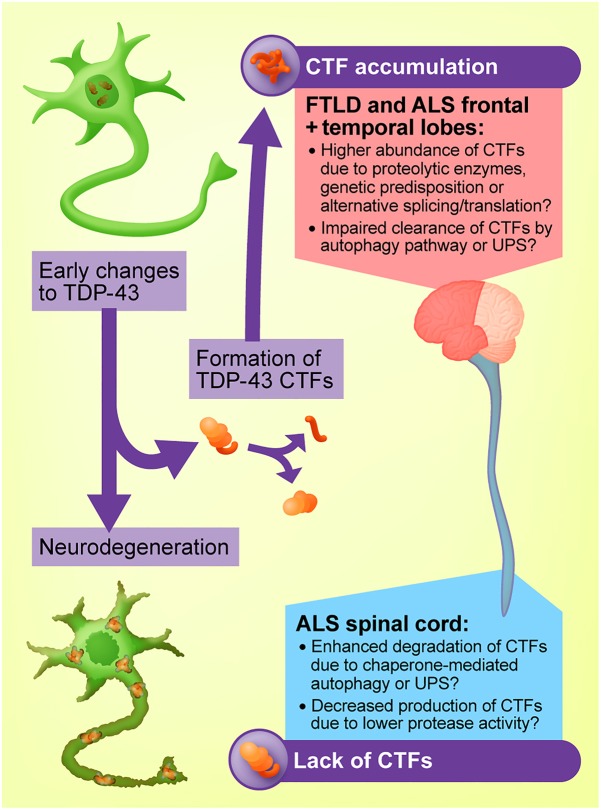
Proposed model for the regional heterogeneity of TDP-43 C-terminal fragments (CTFs). CTFs may accumulate in the brains of people with ALS and FTLD-TDP due to elevated activity of proteases such as caspases and calpains, genetic predisposition, or upregulation of alternative TARDBP transcripts that produce a truncated protein. The lack of CTFs in the ALS spinal cord may arise from mechanisms including enhanced CTF clearance by chaperone-mediated autophagy or the ubiquitin-proteasome system (UPS).

The mechanism through which TDP-43 drives neurodegeneration may differ between the brain and the spinal cord. A prime example of this is the pathological acetylation of TDP-43, which has been detected in the ALS spinal cord but not the brain, likely due to removal of the targeted lysine residues in the N-terminal domain upon cleavage of TDP-43 in the brain (Cohen et al., 2015). The evidence from cell and animal models reviewed above strongly suggests that TDP-43 CTFs are not responsible for neurodegeneration in the brains of people with ALS and FTLD. However, differences in the burden of TDP-43 CTFs across the human CNS is nonetheless interesting as it points to cell-type-specific differences in the way that the TDP-43 protein is modified, which may ultimately determine the cellular effects of TDP-43. It is possible that differences in the ability of distinct groups of cells in the brain and spinal cord to cleave TDP-43, or to clear the resultant fragments, represents a molecular mechanism underpinning the varying abundance of CTFs across the CNS (see Figure 2). In the case of ALS and FTLD, the elevated levels of CTFs in the brain may arise from increased activation of proteases responsible for TDP-43 cleavage, translation of genes that promote cleavage or upregulation of transcripts that produce a truncated TDP-43 protein. By the same principle, it is possible that the relative absence of TDP-43 CTFs in the ALS spinal cord is due to enhanced activity of degradation pathways specific to particular TDP-43 fragments, or decreased activity of the proteases responsible for CTF formation.

Neurons are particularly diverse cells with highly specialized functions. With regard to motor neurons, subtypes vary dramatically in terms of neurotransmitter secretion, excitability and cytoarchitecture (Stifani, 2014), as well as the molecules that regulate cellular stress tolerance (Simandi et al., 2018). Factors such as these may influence cellular responses to TDP-43 and protein accumulation in general. Epigenetic mechanisms also differ according to cell type and CNS sub-region (Davies et al., 2012) and may underlie the regional heterogeneity of CTF distribution by regulating the silencing and transcription of different genes responsible for the formation or degradation of TDP-43 CTFs. Indeed, DNA methylation patterns vary greatly between motor neurons of the cortex and spinal cord in tissue samples from both people with ALS and healthy controls (Chestnut et al., 2011), which could potentially allow TDP-43 CTFs to accumulate in select groups of cells.

Experimental data also indicate that alterations to TDP-43, such as its cleavage into CTFs, may proceed in a cell-type-specific manner. For instance, in people with inclusion body myopathy, an inflammatory muscle disease, TDP-43 is ubiquitinated and translocated to the cytoplasm of the muscle cells but does not appear to be cleaved into CTFs (Weihl et al., 2008). In cell culture, CTF-25 is more prone to aggregation and less readily degraded when transfected into NSC-34 cells in comparison with a muscle cell line (Cicardi et al., 2018). These studies suggest that the formation and aggregation of TDP-43 CTFs is a CNS-specific phenomenon. It has recently been demonstrated that the specific intracellular environment can influence the conformation of α-synuclein and its biological actions, ultimately creating distinct α-synuclein strains that distinguish dementia with Lewy bodies from multiple system atrophy (Peng et al., 2018). This may also hold true for TDP-43, whereby the unique molecular composition of the cell determines how the TDP-43 protein is modified, impacting its resultant biochemical actions.

The distinct features of certain cell populations may also confer vulnerability to TDP-43 pathology. This was recently demonstrated in mice, whereby intra-cortical injection of insoluble protein extracted from FTLD-TDP brains led to a selective spread of pathology to deeper structures that have previously been shown to be affected in the FTLD-TDP brain, such as the nucleus accumbens and basolateral amygdala (Porta et al., 2018). Determining how the distinct gene and protein profiles of various classes of neurons renders certain populations susceptible to TDP-43 pathology or specific post-translational TDP-43 modifications may assist in understanding the complex biology of TDP-43 in diseases such as ALS and FTD that are characterized by dramatic genetic, neuropathological and phenotypic heterogeneity (Simon et al., 2014), and elucidate protective molecular and cellular factors for therapeutic targeting.

## Final Remarks and Conclusion

Amyotrophic lateral sclerosis is a rapidly progressing and debilitating disease, and currently available treatments offer only modest benefits. Riluzole may extend survival by 2–3 months, potentially via attenuation of glutamate excitotoxicity (Bellingham, 2011; Miller et al., 2012). The more recently available edaravone, a free radical scavenger, appears to have a small impact on lifespan and quality of life for a select population of people living with ALS, with results from phase II clinical trials not always reaching significance (Sawada, 2017). Pharmaceutical interventions for FTD are equally disappointing, with no treatments available to alter the rate of disease progression, and the repurposing of existing anti-psychotic medications showing limited efficacy in symptom management (Tsai and Boxer, 2016). TDP-43 pathology has dramatic impacts on the CNS, triggering a multitude of cellular alterations and disease cascades in both neurons and glial cells. To develop new disease-modifying treatments targeted at the underlying pathology, it is critical that research attention is focused on changes to TDP-43-affected cells that have a clear, causal connection to disease.

TDP-43 CTFs are considered a pathological hallmark in the brains of people with ALS and FTLD-TDP. However, these fragments are rarely detected in the ALS spinal cord, despite the dramatic death of lower motor neurons. Exogenous expression of these fragments in cultured cells has provided mixed results and whether CTFs are more toxic to cells than full-length TDP-43 remains debateable. Critically, transgenic animals expressing TDP-43 CTFs do not exhibit a robust ALS-like phenotype of motor dysfunction, and only display mild deficits that potentially resemble broader aspects of neurodegeneration, but not specifically ALS or FTD, in aged animals. Given the low-level detection of these fragments in ALS cerebrospinal fluid (Ding et al., 2015) and FTD plasma (Foulds et al., 2009), further investigation of their efficacy as a biomarker of disease is warranted. However, our evaluation of evidence across cell and animal models and human post-mortem tissue illustrates that TDP-43 CTFs are unlikely to be a primary cause of neurodegeneration in ALS and FTD. For this reason, therapeutics specifically targeted against TDP-43 CTFs are unlikely to modify disease, and further investigation of other potential disease-modifying strategies is warranted.

## Author Contributions

BB and AW conceived and wrote the manuscript and compiled the tables.

## Conflict of Interest Statement

The authors declare that the research was conducted in the absence of any commercial or financial relationships that could be construed as a potential conflict of interest.
